# Supplementary material for: Role of Residence Area on Diet Diversity and Micronutrient Intake Adequacy in Urban and Rural Costa Rican Adolescents
Source: Nutrients. 2022 Dec 1;14(23):5093. doi: 10.3390/nu14235093 (PMC9738538; doi:10.3390/nu14235093)
Supplement: Supplementary file 1 [file nutrients-14-05093-s001.zip › nutrients-2070862-supplementary.pdf]

nutrients

MDPI

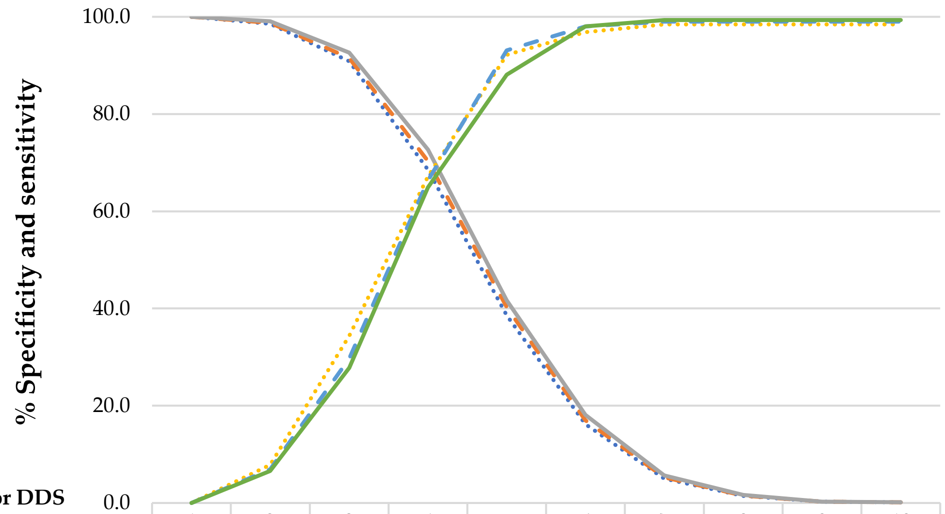

Cut-off points for DDS	1	2	3	4	5	6	7	8	9	10
Specificity (%) MAR 0.65	100.0	98.5	90.8	68.6	38.6	16.2	5.0	1.5	0.3	0.1
Specificity (%) MAR 0.70	100.0	98.7	91.5	70.3	40.3	17.0	5.3	1.5	0.3	0.1
Specificity (%) MAR 0.75	100.0	99.1	92.7	72.7	41.7	18.1	5.7	1.6	0.3	0.1
Sensitivity (%) MAR 0.65	0.0	7.8	34.4	67.2	92.2	96.9	98.4	98.4	98.4	98.4
Sensitivity (%) MAR 0.70	0.0	6.9	29.7	66.3	93.1	98.0	99.0	99.0	99.0	99.0
Sensitivity (%) MAR 0.75	0.0	6.6	27.8	64.9	88.1	98.0	99.3	99.3	99.3	99.3

Figure S1. Sensitivity and specificity of different DDS cut-off points with MAR cut-off points ranging from 0.65 to 0.75, performed as sensitivity analysis for day one of 3-day food record. [1]Specificity: identifies nutritionally appropriate diets as adequate. [2]Sensitivity: identifies nutritionally inappropriate diets as inadequate. DDS: Diet Diversity Score; MAR: Mean Adequacy Ratio.

Table S1. Diet Diversity Score (DDS) and proportion of participants reaching the minimum DDS [1] (*n* = 818), performed as sensitivity analysis for day one of 3-day food record.

Characteristic	Diet Diversity Score (DDS)			Participants reaching the minimum DDS	
	**Mean**	**SD**	***p-value*[2]**	**n (%)**	***p-value*[2]**
Overall	4.10	1.40		537 (65.7)	
Sex					
Female	4.08	1.45	0.719	330 (63.5)	0.082
Male	4.12	1.31		207 (69.5)	
Residence area					
Urban	3.99	1.38	0.043	252 (61.3)	0.009
Rural	4.20	4.41		285 (70.0)	
Socioeconomic status					
Low	4.08	1.37	0.236	174 (66.1)	0.939
Middle	4.07	1.36		211 (64.9)	
High	4.15	1.49		152 (66.1)	
Nutritional status					
Non overweight	4.08	1.47	0.409	349 (63.3)	0.046
Overweight/obesity	4.12	1.27		188 (70.4)	

[1]Consumption of at least 4 out of 10 food groups. [2]*p-values* < 0.05 are statistically significant and were determined using the chi-square, Student's t, or ANOVA tests.

Table S2. Food group consumption of Costa Rican adolescents, according to Diet Diversity Score (DDS) and residence area, performed as sensitivity analysis for day one of 3-day food record.

**Food group[1] (g/d)**	**Diet Diversity**		*p-value*[2]	**Residence Area**		*p-value*[2]
	**DDS <4 (*n*=281)**	**DDS ≥4 (*n*=537)**		**Urban (*n*=411)**	**Rural (*n*=407)**	
Starchy staples	476.6 ± 282.8	486.8 ± 267.6	0.422	441.5 ± 266.2	525.5 ± 273.2	< 0.0001
Milk and milk products	89.6 ± 179.5	182.9 ± 229.1	< 0.0001	170.1 ± 235.2	131.4 ± 197.2	0.019
Pulses	66.4 ± 99.7	129.3 ± 120.4	< 0.0001	91.6 ± 109.9	124.0 ± 122.7	< 0.0001
Flesh foods	79.7 ± 117.7	121.0 ± 105.0	< 0.0001	107.0 ± 114.1	106.6 ± 108.4	0.835
Other fruits	63.8 ± 192.5	196.4 ± 282.2	< 0.0001	147.2 ± 263.9	154.5 ± 261.4	0.614
Other vegetables	5.7 ± 42.6	54.6 ± 132.1	< 0.0001	27.4 ± 86.5	48.3 ± 132.7	0.030
Eggs	7.2 ± 25.1	25.3 ± 37.0	< 0.0001	18.8 ± 34.7	19.3 ± 34.2	0.462
Other vitamin A-rich F&V	3.8 ± 28.9	42.0 ± 136.3	< 0.0001	28.7 ± 114.4	29.1 ± 112.1	0.495
Dark green leafy vegetables	0.3 ± 4.7	21.6 ± 110.0	< 0.0001	4.8 ± 29.7	23.9 ± 123.0	< 0.0001
Nuts and seeds	0.5 ± 4.4	3.7 ± 17.5	< 0.0001	2.0 ± 10.7	3.3 ± 17.4	0.188

[1]Values are means ± SD. [2]*p-values* < 0.05 are statistically significant and were determined using the Wilcoxon test. DDS: Diet Diversity Score; F&V: Fruits and vegetables.

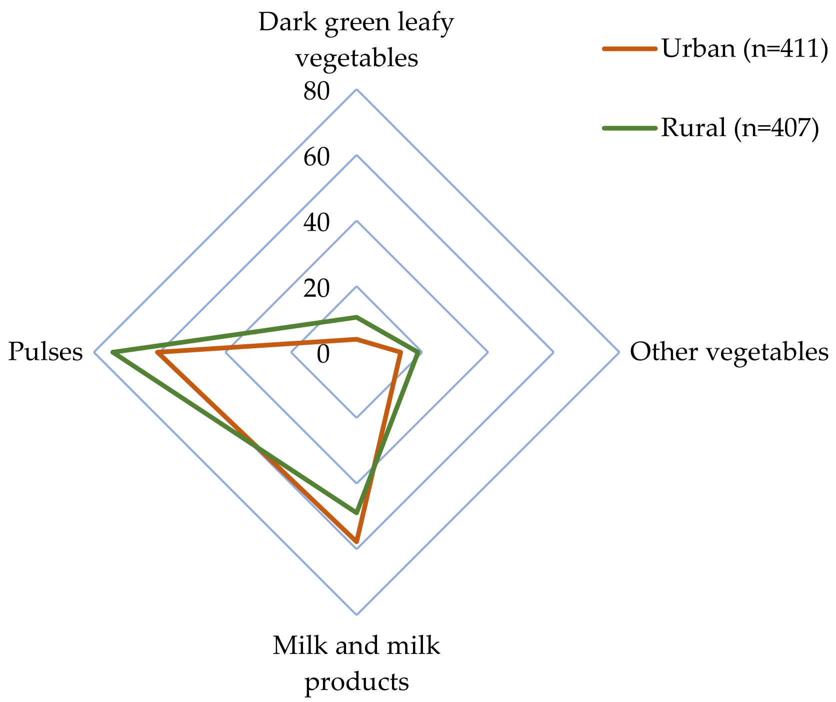

Figure S2. Proportion of participants consuming at least 15 g of each food group over one-day food record, according to residence area, performed as sensitivity analysis for day one of 3-day food record. Only food groups with significant differences between urban and rural areas are presented.

Table S3. Bivariate and multivariate logistic regression analysis: Sociodemographic variables related to having a diverse diet, according to Diet Diversity Score (DDS) (n = 818), performed as sensitivity analysis for day one of 3-day food record.

Variable	Bivariate Analysis			Multivariate Analysis[1]		
	OR	95% CI	*p-value*	Adj OR	95% CI	*p-value*
Sex						
Women	0.76	0.56 – 1.03	0.082	0.76	0.56 – 1.04	0.091
Age	0.99	0.91 – 1.08	0.824	0.99	0.91 – 1.08	0.788
Residence area						
Rural	1.47	1.22 – 2.20	0.009	1.53	1.12 – 2.08	0.007
Socioeconomic status						
Middle	0.95	0.67 – 1.33	0.754	0.98	0.69 – 1.40	0.931
High	1.00	0.69 – 1.45	0.986	1.13	0.76 – 1.69	0.536
Nutritional status						
Overweight/obesity	1.38	1.01 – 1.89	0.046	1.36	0.99 – 1.88	0.055
Constant	-	-	-	1.94	0.49 – 7.63	0.034

[1]Overall model test *p-value* = 0.024. Goodness-of-fit test *p-value* = 0.701. Correct predictions: 65.6%.

Table S4. Mean Nutrient Adequacy Ratio (NAR), according to Diet Diversity Score (DDS), performed as sensitivity analysis for day one of 3-day food record.

Nutrients	Overall (n=818)		DDS <4 (*n*=281)		DDS ≥4 (*n*=537)			r^1	*p-value*
	Mean	SD	Mean	SD	Mean	SD	*p-value*[2]		
Calcium	0.519	0.290	0.433	0.285	0.564	0.283	< 0.0001	0.283	< 0.0001
Iron	0.955	0.129	0.911	0.182	0.979	0.080	< 0.0001	0.250	< 0.0001
Zinc	0.817	0.219	0.721	0.249	0.867	0.183	< 0.0001	0.344	< 0.0001
Vitamin C	0.817	0.279	0.711	0.329	0.873	0.231	< 0.0001	0.314	< 0.0001
Thiamin	0.955	0.130	0.919	0.175	0.973	0.093	< 0.0001	0.204	< 0.0001
Riboflavin	0.924	0.163	0.867	0.216	0.954	0.117	< 0.0001	0.291	< 0.0001
Niacin	0.937	0.143	0.889	0.192	0.962	0.102	< 0.0001	0.242	< 0.0001
Vitamin B6	0.890	0.185	0.797	0.233	0.939	0.130	< 0.0001	0.390	< 0.0001
Folate equivalents	0.954	0.138	0.909	0.193	0.978	0.089	< 0.0001	0.229	< 0.0001
Cobalamin	0.897	0.207	0.828	0.259	0.933	0.163	< 0.0001	0.260	< 0.0001
Vitamin A	0.695	0.301	0.606	0.325	0.742	0.278	< 0.0001	0.232	< 0.0001
MAR	0.851	0.138	0.781	0.166	0.888	0.103	< 0.0001	0.412	< 0.0001

[1]Spearman's rank correlation coefficients (*r*) were calculated between each NAR/MAR value and the DDS for the whole sample. [2]*p-values* < 0.05 are statistically significant and were determined using the Wilcoxon test. NAR: Nutrient Adequacy Ratio; MAR: Mean Adequacy Ratio; DDS: Diet Diversity Score.

Table S5. Proportion of participants reaching Nutrient Adequacy Ratio (NAR) ≥ 0.70, according to Diet Diversity Score (DDS) and residence area), performed as sensitivity analysis for day one of 3-day food record.

Nutrient	Overall[1] (*n*=818)	DDS < 4 (*n*=281)	DDS ≥ 4 (*n*=537)		Urban (*n*=411)	Rural (*n*=407)	
	n (%)	*n* (%)	*n* (%)	*p-value*[2]	*n* (%)	*n* (%)	*p-value*[2]
Calcium	242 (29.6)	58 (20.6)	184 (34.3)	< 0.0001	125 (30.4)	117 (28.8)	0.602
Iron	768 (93.9)	247 (87.9)	521 (97.0)	< 0.0001	375 (91.2)	393 (96.6)	0.001
Zinc	580 (70.9)	151 (53.7)	429 (79.9)	< 0.0001	275 (66.9)	305 (74.9)	0.011
Vitamin C	592 (72.4)	165 (58.7)	427 (79.5)	< 0.0001	296 (72.0)	296 (72.7)	0.821
Thiamin	764 (93.4)	245 (87.1)	519 (96.7)	< 0.0001	374 (91.0)	390 (95.8)	0.005
Riboflavin	729 (89.1)	225 (80.1)	504 (93.9)	< 0.0001	352 (85.6)	377 (92.6)	0.001
Niacin	707 (86.4)	211 (75.1)	496 (92.4)	< 0.0001	344 (83.7)	363 (89.2)	0.022
Vitamin B6	687 (84.0)	192 (69.3)	495 (92.2)	< 0.0001	328 (79.8)	359 (88.2)	0.001
Folate equivalents	762 (93.2)	242 (86.1)	520 (96.8)	< 0.0001	373 (90.8)	389 (95.6)	0.006
Cobalamin	694 (84.8)	209 (74.4)	485 (90.3)	< 0.0001	339 (82.5)	355 (87.2)	0.059
Vitamin A	450 (55.0)	125 (44.5)	325 (60.5)	< 0.0001	223 (54.3)	227 (55.8)	0.663
MAR	717 (87.7)	213 (75.8)	504 (93.9)	< 0.0001	348 (84.7)	369 (90.7)	0.009

[1]Values are frequencies (%) unless otherwise indicated. [2]*p-values* < 0.05 are statistically significant and were determined using the chi-square test. NAR: Nutrient Adequacy Ratio; MAR: Mean Adequacy Ratio; DDS: Diet Diversity Score.

Table S6. Mean Nutrient Adequacy Ratio (NAR) and Diet Diversity Score (DDS), comparing days of the 3-day food record, performed as sensitivity analysis.

Nutrients	Overall (*n*=818)		Day 1 (*n*=818)		Day 2 (*n*=818)		
	Mean	SD	Mean	SD	Mean	SD	*p-value*[1]
Calcium	0.506	0.292	0.519	0.290	0.492	0.292	0.049
Iron	0.962	0.130	0.955	0.129	0.968	0.131	< 0.0001
Zinc	0.802	0.235	0.817	0.219	0.788	0.249	0.279
Vitamin C	0.851	0.275	0.817	0.279	0.885	0.268	< 0.0001
Thiamin	0.960	0.131	0.955	0.130	0.965	0.132	< 0.0001
Riboflavin	0.932	0.168	0.924	0.163	0.940	0.173	< 0.0001
Niacin	0.946	0.148	0.937	0.143	0.954	0.151	< 0.0001
Vitamin B6	0.894	0.199	0.890	0.185	0.898	0.213	< 0.0001
Folate equivalents	0.962	0.133	0.954	0.138	0.969	0.128	< 0.0001
Cobalamin	0.927	0.187	0.897	0.207	0.957	0.159	< 0.0001
Vitamin A	0.690	0.306	0.695	0.301	0.686	0.310	0.984
MAR	0.857	0.137	0.851	0.138	0.864	0.135	0.021
DDS	4.131	1.417	4.097	1.400	4.165	1.433	0.239

[1]*p-values* < 0.05 are statistically significant and were determined using the Wilcoxon test. NAR: Nutrient Adequacy Ratio; MAR: Mean Adequacy Ratio; DDS: Diet Diversity Score.
